# Seasonal and Simultaneous Cleistogamy in Rostrate Violets (*Viola*, subsect. *Rostratae*, Violaceae)

**DOI:** 10.3390/plants10102147

**Published:** 2021-10-10

**Authors:** Ali Ammarellou, Justyna Żabicka, Aneta Słomka, Jerzy Bohdanowicz, Thomas Marcussen, Elżbieta Kuta

**Affiliations:** 1 Research Institute of Modern Biological Techniques, University of Zanjan, Zanjan 45371-38791, Iran; amarlou@znu.ac.ir; 2 Department of Plant Cytology and Embryology, Institute of Botany, Faculty of Biology, Jagiellonian University in Kraków, 9 Gronostajowa St., 30-387 Cracow, Poland; aneta.slomka@uj.edu.pl (A.S.); elzbieta.kuta@uj.edu.pl (E.K.); 3 Department of Plant Cytology and Embryology, Faculty of Biology, University of Gdańsk, 59 Wita Stwosza St., 80-308 Gdańsk, Poland; jerzy.bohdanowicz@ug.edu.pl; 4 Department of Biosciences, Centre for Ecological and Evolutionary Synthesis (CEES), University of Oslo, P.O. Box 1066 Blindern, NO-0316 Oslo, Norway; thmsmrcssn@gmail.com

**Keywords:** *Viola* sp., floral structure, flower types, floral meristems, environmental factors, CH/CL ratio modification

## Abstract

The special mixed reproductive system, i.e., the ability of an individual plant to develop both open, chasmogamous (CH) flowers adapted to cross-pollination and closed, cleistogamous (CL) flowers with obligate self-pollinating, is a common phenomenon in *Viola* L. In most sections of Northern Hemisphere violets, cleistogamy is seasonal, and CH and CL flowers develop sequentially in the season. Non-seasonal cleistogamy (simultaneous) is a rare phenomenon in rostrate violets. In the current study, we focused on modification of the CH/CL mating system in *V*. *caspia* by environmental conditions, resulting in a gradual switch from temporal cleistogamy, occurring in nature, to simultaneous cleistogamy under greenhouse conditions. *V. reichenbachiana* with seasonal cleistogamy was the control for *V. caspia* with the labile seasonal/simultaneous cleistogamy system. In simultaneous cleistogamy, the CH and CL flowers, fruits and seeds developed on an individual plant at the same time on the same branch. The typical difference between CH and CL flowers’ pistils is a straight style ending with a head-like stigma in CH and a curved style in CL adapted to self-pollination. This trait persists in the fruit and seed stages, allowing for easy recognition of fruit of CL and CH flowers in simultaneous cleistogamy. Floral meristems of CH flowers of *V. reichenbachiana* developed on the rhizome at the end of the growing season under short-day conditions and remained dormant until the following season. The CL floral meristems formed under long-day conditions on elongating lateral branches in the upper leaf axils. The daily temperature influenced the variable CH/CL ratio of *V. caspia* in nature and greenhouse conditions. Regulation of the CL/CH flower ratio by modifying environmental factors is important for basic research on genetic/epigenetic regulation of cleistogamy and for practical use to produce genetically stable lines of economically important species via CL seeds.

## 1. Introduction

Cleistogamy is a special sexual breeding system in which permanently closed, self-pollinated flowers are formed. This phenomenon has intrigued botanists since the 19th century. The term was introduced by Kuhn [1], who observed bud-like flowers that never opened but produced fruits and named them as cleistogamous (CL) flowers. Later, Darwin [2] added that in a CL species, the CL flowers may be the only flower type produced by a plant or may accompany open, insect-pollinated chasmogamous (CH) flowers developing on the same plant. The transition between CH and CL flowers could be gradual, and intermediate flowers with a reduced corolla size and curved pistil style that are facultatively autogamous, termed ‘semi-cleistogamous’ (SEMCL), are produced by numerous *Viola* species [3,4]. This special mixed breeding system was found in a variety of plant taxa. It is widespread in angiosperms, occurring in ca. 50 families of monocots and dicots, and may have evolved 34 to 41 times [3,4,5]. Cleistogamy might be beneficial in unfavorable environmental and resource-poor conditions where the abundance of pollinators is drastically reduced, and self-pollinated CL flowers assure reproductive success [5,6,7,8,9,10]. This breeding system is plastic, and the frequency of CL and CH flowers on an individual plant and the timing of flowering may vary depending on environmental conditions as adaptation to heterogenous habitats [11].

The interest in cleistogamy research is mostly focused on the morphology of CH and CL flowers, factors inducing CL flowering and the genetic and molecular basis of the development of both flower types. Different heterochronic processes are involved in the formation of small, unopened CL flowers in *Viola odorata* (sect. *Viola*), which appear to end development prematurely relative to CH [12,13,14]. The CH flowers of *V. odorata* develop in response to short days and the CL flowers in response to long days [15 and references cited therein]. Induction of CL flower development is important in crops (rye, barley, soya) and medicinal and ornamental plants to produce genetically uniform selected lines through self-pollination. The physiology, genetic/epigenetic regulation and molecular mechanisms of cleistogamy still need extensive investigation, although several papers have been published over the last decades [15,16,17,18,19,20,21,22,23]. 

Cleistogamy is relatively common in *Viola*, the largest genus in the family Violaceae, comprising ca. 600 species distributed mainly in the Northern Hemisphere [24,25], which has been used as a good model for studying cleistogamy [5,11,19,21,22,26,27,28]. Most North Hemisphere *Viola* sections are characterized by seasonal cleistogamy in which CH and CL flowers do not develop simultaneously on an individual plant, but temporal production of CH and CL flowers is strongly influenced by environmental factors such as light quantity, canopy cover, photoperiod, temperature, soil pH and moisture [11]. 

Both species studied herein, *V. reichenbachiana* Jordan ex Bor. and *V. caspia* (Rupr.) Freyn, are caulescent perennials belonging to the palaeotetraploid sect. *Viola* subsect. *Rostratae* (Kupffer) W. Becker [29,30]. *Viola reichenbachiana* is distributed in western Eurasia (from central Spain and the British Isles east to Estonia and Greece, northern Turkey, the Caucasus and northwestern Iran) [31]. *Viola caspia*, an important ornamental and medicinal plant in northern and northwestern Iranian folklore, is distributed from Turkey in Europe and Crimea eastwards to northern Iran [30,32,33]. The two species are sympatric in northern Iran and in other regions; they have an overlapping ecology and share one genome (2n = 20). In this study, *V. reichenbachiana* with seasonal cleistogamy was included as the biological background of the flowering process in *Rostratae* violets vs. *V. caspia* with the labile seasonal/simultaneous cleistogamy system.

The main focus of the research was to test the influence of variable experimental conditions on the flowering timing of the CH/CL mating system in *V. caspia* with the implementation of additional research on constant floral traits allowing to distinguish CH and CL flowers and fruits at different stages of development in non-temporal cleistogamy. 

## 2. Results

### 2.1. The Location of CH and CL Flower Meristems in Viola reichenbachiana 

**Figure 1:**
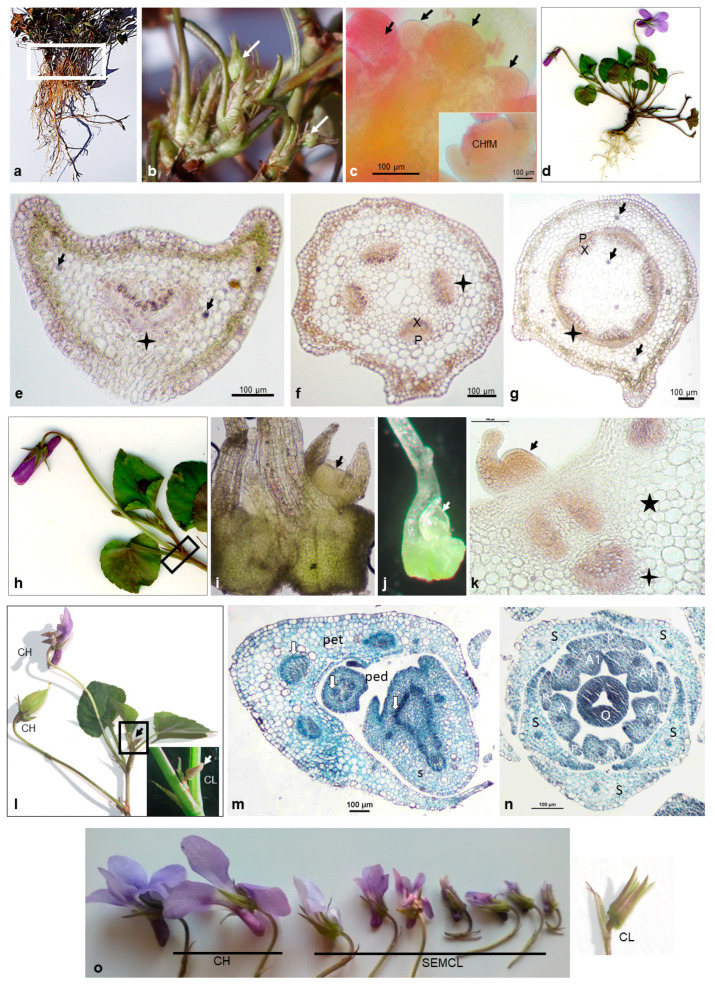
*Viola reichenbachiana*. (**a**–**d**) The cycle of CH flowering. (**a**) Plant at the end of season (January). Marked region on rhizome apices indicates location of CH flower meristems/buds which will develop next spring. (**b**) CH buds (arrows) in the region marked in (**a**). (**c**) CH meristems (arrows) in the regions marked in (**a**), single CH meristem inserted. (**d**) Plant at CH flowering (April/May). (**e**–**g**) Cross-sections of CH petiole (**e**), peduncle (**f**) and stem (**g**). Vascular bundles (stars), xylem (X), phloem (P) and druses (arrows). (**h**) Stage of CH flowering, region with CL (cleistogamous) meristems/buds marked. (**i**,**j**) CL meristems (arrow) in the region marked in (**h**). (**k**) Cross-section of the region marked in (**h**), CL meristem (arrow) with a part of petiole tissue (asterisk) and vascular bundle (star). (**l**) Part of plant (May) with CH flower, CH capsule and CL bud-like flower (arrow), inserted CL flower. (**m**) Cross-section of marked region in (**l**), petiole (pet), peduncle (ped), stem (S) and vascular bundles (arrows). (**n**) Cross-section of CL flower, five anthers (A), two evidently larger (A1), five sepals (S), five reduced petals (P) and ovary (O). (**o**) Flower types: CH—chasmogamous; SEMCL—semi-cleistogamous; CL—cleistogamous.

At the end of the growing season (starting from November/December) under short-day conditions, the meristems/buds of CH flowers were observed on the rhizome (Figure 1a–c). Dormant buds survived the winter and developed into CH flowers the following season (Figure 1d). 

Meristems of CL flowers developed at CH flowering. To determine the location of CL meristems, the stem, petiole and CH peduncle anatomy was analyzed. All organs had characteristic wings; the vascular system was organized as a collateral bundle. In petioles, abaxial xylem and adaxial phloem formed an arched bundle (Figure 1e). In a peduncle, four vascular bundles (Figure 1f), and in stem, several bundles (Figure 1g) with abaxial phloem and adaxial xylem were separated by parenchyma tissue. 

The CL floral meristems were formed on elongated lateral branches in the upper leaf axils under long-day conditions in the angles between the lateral stem, the petiole and the peduncle of CH flowers (Figure 1h–m).

### 2.2. Differences in the Floral and Fruit Morphology between CH and CL Flowers

**Figure 2:**
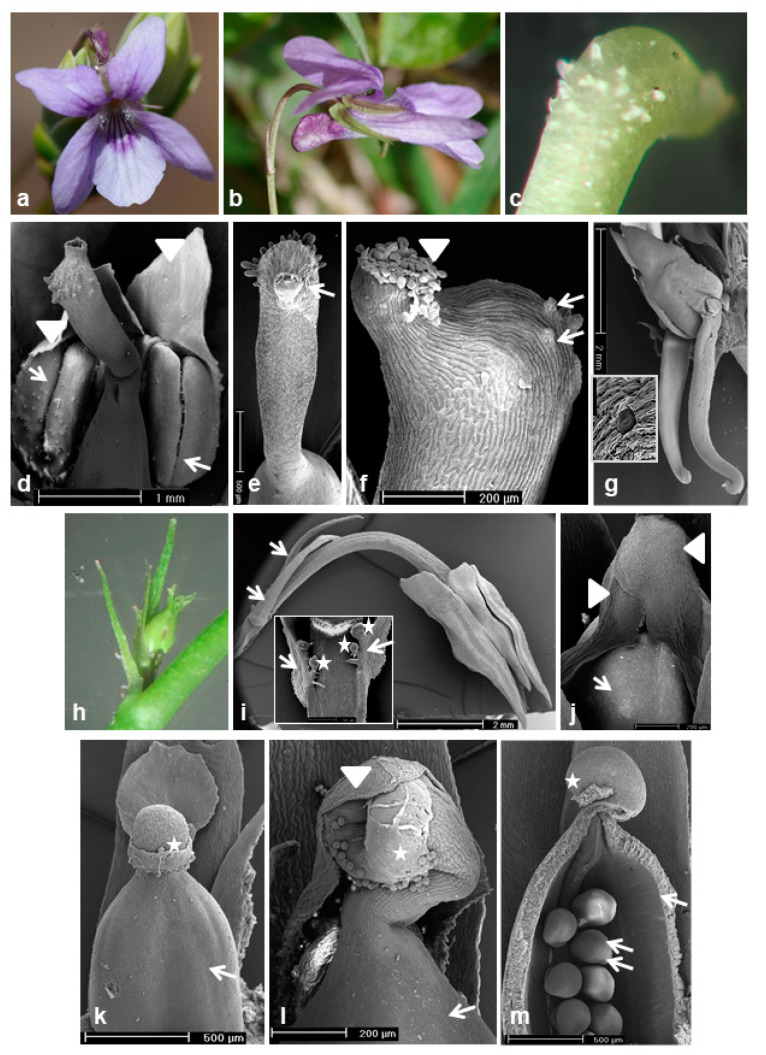
*Viola reichenbachiana*: (**a**–**g**) floral structure of CH and (**h-m**) CL flowers. (**a**) Face view of flower. (**b**) Lateral view of flower, spur of flower visible. (**c**) Style with head-like hairy/papillous stigma of pistil. (**d**) Pistil and anthers (arrows) with appendages (arrowheads). (**e**,**f**) Style with hairy/papillous stigma (**e**, arrow), stigma hole filled with pollen grains (**f**, arrowhead), hairs/papillae on head-like stigma (**f**, arrows). (**g**) Long anther appendages (nectaries) inserted into the spur in CH flower, stomata of nectaries (inserted). (**h**,**i**) CL flower, glands (asterisk) located close to bracts (arrows) inserted in (**i**). (**j**) Appendages of two reduced anthers (arrowheads) covering stigma, enlarged ovary (arrow). (**k**–**m**) Pistil with enlarged ovary (arrows) and curved style with stigma (asterisks), visible anther appendage (arrowhead) and germinated pollen grains on stigma (**l**), ovules in open ovary (**m**, doubled arrows). (**d**–**g**,**i**–**m**) SEM micrographs.

**Figure 3:**
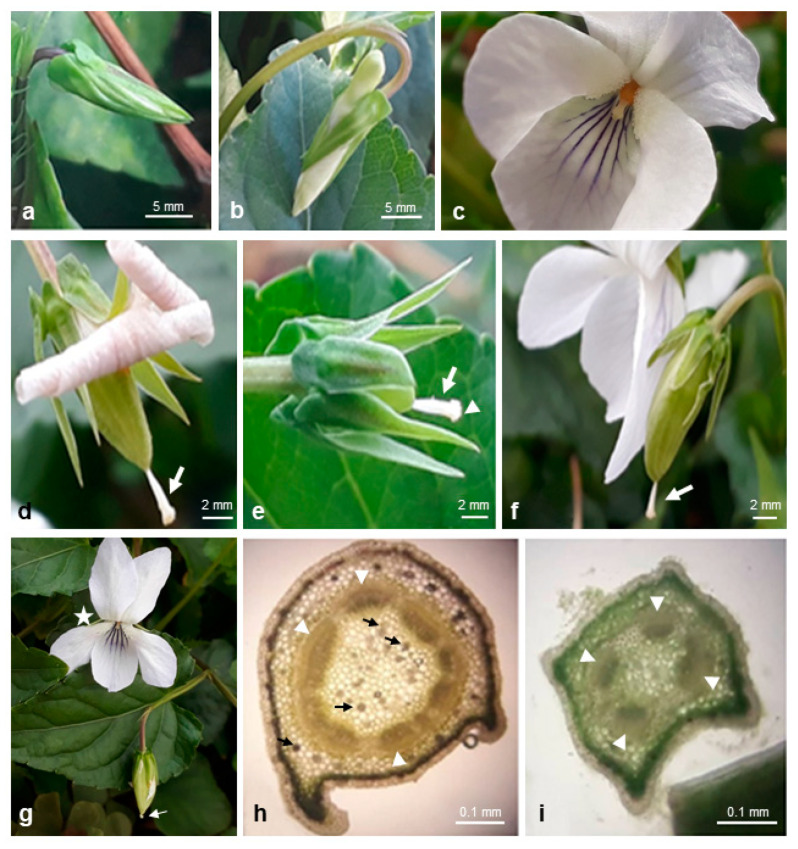
*Viola caspia* under greenhouse conditions. CH flower developmental stages. (**a**,**b**) Buds. (**c**) Face view of flower. (**d**–**f**) Stages of fruit development, enlarged ovary, straight pistil style (arrows) ending with head-like hairy/papillous stigma (arrowhead in **e**). (**g**) CH flowers (asterisk) and fruit of CL flowers; note curved style of pistil (arrow). (**h**,**i**) Cross-section of stem (**h**) and CH peduncle (**i**), vascular bundles (arrowheads), druses in the parenchyma of pith and cortex (arrows).

Deep violet CH flowers with a spur longer than the sepal appendages of *V. reichenbachiana* (Figure 2a,b) and white CH flowers of *V. caspia* (Figure 3a–c) both exhibited characteristic structures for the *Rostratae* subsection. Their perianth comprised five green sepals, five petals: one lower petal (spurred) with lines as the guide for pollinators, two lateral petals facing downwards and two upper petals slightly tilted (Figure 2a and Figure 3c). The pistils of the CH flowers of both species had a straight style ending with a hairy/papillous head-like apex with a beak. The stigma with a hole in it was located at the apex of the beak (Figure 2c–f and Figure 3d). Five filamentless stamens with anther appendages tightly covering the style of the pistil (Figure 2d) were morphologically differentiated. Two had long nectariferous appendages with stomata in the epidermis (Figure 2g) projecting into the spur where the produced nectar flows.

After pollination, the enlarged ovary developed into a fruit (capsule) filled with seeds. At this stage, the erect style of the pistil ending with the stigma was still visible as a characteristic feature, allowing to distinguish the CH flower from the CL one with a curved style (Figure 3e,f). 

**Figure 4:**
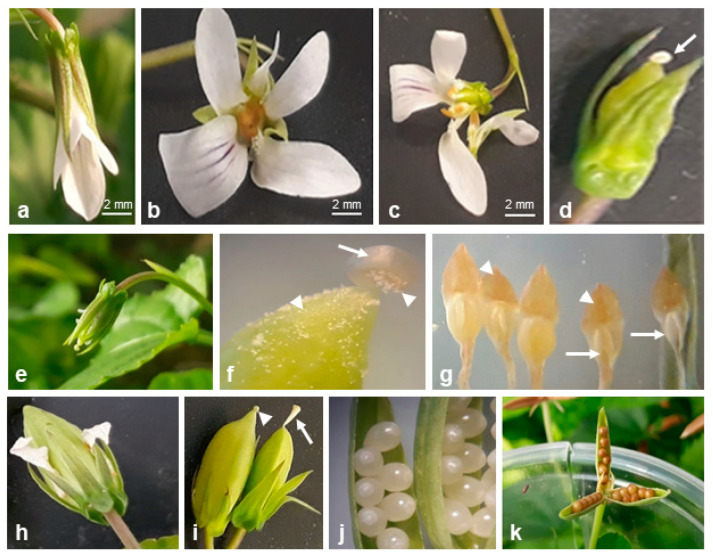
*Viola caspia* under greenhouse conditions. SEMCL and CL flowers and fruits. (**a**–**d**) SEMCL flowers at different stages of development (**a**–**c**), enlarged ovary, curved style of pistil (arrow) (**d**). (**e**–**g**) CL bud-like flower (**e**), enlarged ovary, curved style of pistil (arrow), pollen grains on stigma and ovary (arrowheads) (**f**), five stamens with reduced anthers (arrows) ending orange appendages (arrowheads) (**g**), enlarged ovaries (**h**,**i**); note differences in style of pistil shape, short and curved in CL (arrowhead in (**i**)), straight in CH (arrow in (**i**)). (**j**) Enlarged ovules in CL fruit, (**k**) seeds in CL fruit.

The semi-cleistogamous flowers of both species were smaller than the CH flowers and had all floral elements (Figure 1o and Figure 4a–c), but a curved style of the pistil (Figure 4d). 

Cleistogamous flowers of *V. reichenbachiana* (Figure 1n and Figure 2h,i) and *V. caspia* (Figure 4e) had reduced petals and anthers and a curved style with anther appendages covering the stigma (Figure 2j,k and Figure 4f,g). Pollen grains germinated through the anther wall directly on the stigma (Figure 2l). After self-fertilization, the ovary and ovules increased in size (Figure 2m and Figure 4h–j) and developed into fruits filled with seeds (Figure 4k). During fruiting, the curved pistil style was still visible (Figure 4i, comparison of pistil styles of CH and CL fruits). 

In *V. caspia*, CH and CL flowers and fruits developed simultaneously on an individual plant on the same branch under greenhouse conditions (Figure 3g). The anatomy of the *V. caspia* stem and peduncle was comparable with that of the stem and peduncle of *V. reichenbachiana* (compare Figure 3h,i with Figure 1f,g).

### 2.3. Greenhouse Conditions Switched Seasonal Cleistogamy to Simultaneous in Viola caspia

*Viola reichenbachiana* seasonally developed CH and CL flowers. CH flowering was brief (late April to May/early June, depending on the year), followed by a long CL flowering ending in late November. At the end of CH flowering and at the beginning of CL flower development, SEMCL flowers (intermediate in floral morphology between CH and CL) were produced (Figure 1). *Viola caspia*, similar to *V. reichenbachiana*, developed CH, SEMCL and CL flowers. 

**Figure 5:**
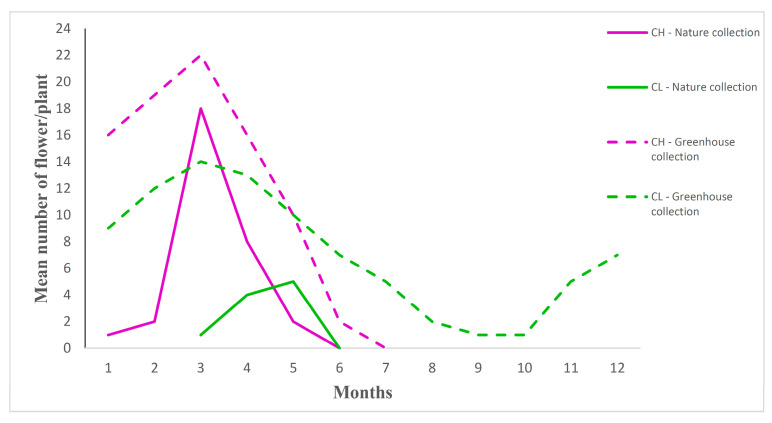
Number of CH and CL flowers per plant of *Viola caspia* developing during a season in Live Collection of Iranian Violets and in greenhouse conditions. N = 20. Five-year average results.

In the condition of Live Collection of Iranian Violets, CH and CL flowering was partly temporally separated (seasonal cleistogamy). The CH flowering time lasted five months, starting in January and ending in May. Abundant CH flowering was observed in March and then the number of flowers per plant decreased. The CL flowering time was evidently shorter (three months), starting in March and ending in May, with the most CL flowers per plant in May (Figure 5, Table S1). After March, the number of CH flowers decreased and CL flowers increased, with the highest CH/CL ratio (18) in March and the lowest in May (0.4) (Table S1).

In greenhouse conditions, CH and CL flowering duration was extended. CH flowering lasted six months (January-June) and CL flowering lasted twelve months (January-December). Abundant CL and CH flowering was observed in March (Figure 5). Both flower morphs developed on a single plant simultaneously during the period from January until June. After this period, CH flowering stopped, whereas CL flower production continued until December. During the whole season, the CH/CL ratio was evidently lower in greenhouse conditions than in the Live Collection of Iranian Violets, with the highest in January (1.77) and the lowest in June (0.28) (Table S2). 

These two habitats differed significantly in terms of mean daily temperature but much less so in the case of humidity (Table S3). The changing temperature throughout the year in the LCIV conditions (mean daily temp. ranged from 0.42 to 25.83 °C; Table S3) had an impact on the CH flower production, and the negative correlation was confirmed (r = −0.62466, 0.02 < *p* < 0.05). Such a correlation was not observed in the greenhouse conditions. In turn, in this temperature-stable conditions (mean daily temp. ranged from 20.4 to 24.4 °C; Table S3), the number of CL flowers was positively correlated with the number of CH flowers (r = 0.8883, *p* ≤ 0.001).

## 3. Discussion

Changed environmental variables in this experimental study, modified the mixed CL/CH reproductive system. The greenhouse conditions, differing from natural conditions, led not only to a change in the flowering time of CH and CL flowers and the CH/CL ratio in *V. caspia*, but also to a gradual switch from seasonal cleistogamy (occurring in nature) to simultaneous CH and CL flower production. There were conspicuous differences in temperature between the Live Collection of Iranian Violets (LCIV) and greenhouse conditions. In LCIV, the daily temperature varied throughout the year (0.42 °C in March, 25.83 °C in September); in the greenhouse, it was more stable and did not drop below 20 °C. The daily temperature influenced the variable CH/CL ratio of *V. caspia* in LCIV and greenhouse conditions. The photoperiod, one of the main factors inducing CH/CL flowering in nature, was the same in the LCIV and greenhouse conditions (artificial light was not supplied in the greenhouse), thus not influencing the differences in the CH/CL ratio in *V. caspia*.

Our results add to the research on the impact of environmental variables on the development of the two flower morphs in cleistogamous species. Following the studies of *Viola pubescens* which clearly indicated that, in nature, seasonally variable environmental factors can drastically modify the mixed breeding system of this species [11], we showed that the experimental manipulation of environmental variables could preferentially induce each bud type. These results open the possibility of experimental modification of the environmental factors influencing the temporal ‘gap’ between CH and CL flowers in species with seasonal cleistogamy, such as *V. caspia*, and indicate that the developmental program of cleistogamous species in nature might not be absolute/directional. The results from the greenhouse conditions clearly show that the daily temperature significantly affected the CH/CL flower ratio, with a clear predominance of CL flowers. This allows for long-term predictions of how global climate change (warming) will affect the CH/CL ratio of species with this reproductive system, which would have an impact on the reduction in population genetic differentiation.

Our results documented constant differences between CH and CL flowers at different stages of their development and at fruiting, allowing for the easy recognition of fruits derived from CH or CL flowers. CH flowers attract insect pollinators and promote cross-pollination and potential genetic diversity within populations [34,35,36,37,38,39,40]. In contrast, obligate self-pollinated CL flowers produce pure lines. For breeders, selecting lines with important characteristics, especially in crops and medicinal and ornamental plants, is crucial. *Viola caspia* is a very variable species in terms of both extreme phenotypic plasticity and habitat ecology (steppe to creek sides to deep beech forests) [41,42]. Its oil of high medicinal value, practically derived from soaking and preserving violet petals in olive or sesame oil, but is not an original seed oil [43]. The most important is pure oil extracted from seeds. For this purpose, the genetically stable seeds of CL flowers are more suitable than CH seeds. The production of high amounts of CL seeds in a growing season allows for the extraction of pure oil from the seeds. 

Distinguishing CH and CL fruits of violets with seasonal cleistogamy in the field is not difficult because both flower types and fruits are temporally separated in the flowering season as in *V. reichenbachiana* and *V. caspia* in nature. In simultaneous cleistogamy, induced in *V. caspia* under greenhouse-controlled conditions, the flowering and fruiting of CH and CL occurred at the same time (on the same plant, on one branch), and the distinction between CH and CL fruits was based on a constant pistil trait (curved style of CL pistil vs. straight in CH) which persists until fruiting. The traits of the CH and CL generative organs of *V. reichenbachiana* and *V. caspia* are not variable and not influenced by environmental conditions (temperate climatic zone vs. subtropical), similar to the stem and peduncle anatomy. 

Modification of CH and CL bud fate by manipulation of environmental variables in experimental conditions opens a new area for further deep insight into the genetic regulation of chasmogamous/cleistogamous systems and could enable the control of outcrossing and self-fertilization in cleistogamous species.

## 4. Materials and Methods

### 4.1. Study Species

Specimens of *V. reichenbachiana*, early dog violet, were grown in an artificial forest in Cracow (Poland; 50°04’42.8” N 19°59’08.9” E). This species with seasonal cleistogamy served as a reference against *V. caspia* with the labile seasonal/simultaneous cleistogamy system.

Plants were observed over several seasons (2014–2017), and plant material was collected for determination of the location of floral meristems and flower structure.

The number of *V. reichenbachiana* specimens used in this study depended on the analysis: for CH flower meristem/buds location, ~10 plants were dug up, and the same plant number was used for CL meristem/buds location. For CH and CL flower morphology and microstructure, 4–5 flowers were collected from different plants, and 3–4 petioles or stem fragments were analyzed for organ anatomy.

Forty specimens of *V. caspia* were randomly collected in their natural habitat in the Gilan-Saravan forest, located 13 km southwest of Sangar and 35 km from Rasht (Iran; 37°04’ N 49°39’ E, 70 m a.s.l.), and were transported to the Live Collection of Iranian Violets (LCIV) at the Research Institute of Modern Biological Techniques, University of Zanjan, Iran, located 150 km from Saravan forest (36°41’ N 48°23’ E, 1579 m a.s.l.).

### 4.2. Location of Floral Meristems in V. reichenbachiana

Chasmogamous floral meristems/buds were collected in December/January 2015/2016. The plants were gently dug from the soil to avoid damaging the root system and rhizome apices and inspected for CH buds using a stereoscopic microscope (OPTA-TECH, Warsaw, Poland). Rhizome apices were fixed in a mixture of ethanol (96%; Avantor Performance Materials Poland S.A., Gliwice, Poland) and glacial acetic acid (3:1 *v*/*v*; Avantor Performance Materials Poland S.A., Gliwice, Poland) for 24 h and then stained with acetic orcein (Honeywell Fluka™, Charlotte, NC, USA) and gently squashed to observe CH meristems. 

For CL flower meristems, the leaf axis regions of floriferous stems were collected at CH flowering in April/May 2016 and 2017, fixed as described above and then stained in 1% acetocarmine (Sigma-Aldrich, Saint Louis, MO, USA) for 48 h, gently squashed or cross-sectioned by hand.

The localization of CH and CL floral meristems/buds in *V. caspia* has not been studied or documented because it is the same as that for *V. reichenbachiana*. Both species belong to the subsect. *Rostratae* of *Viola* section, in which floral features are conservative. 

### 4.3. Morphology of CH and CL Flowers

All stages of CH and CL flowers of both species were analyzed during the season, and the differences in pistil shape, style and stamens between CH and CL flowers were documented with a stereoscopic microscope (Nikon SMZ1, Tokyo, Japan). Photographs were taken with an Olympus microscope (Olympus CX31, Tokyo, Japan) under 40× and 400× magnifications. 

### 4.4. Microstructure of CH and CL Flowers by SEM

For floral trait analyses of *V. reichenbachiana*, CH and CL flowers were fixed in acetic acid–alcohol (FAA) (40% formaldehyde, glacial acetic acid and 70% ethanol, 0.5/0.5/9, v/v/v; Avantor Performance Materials Poland S.A., Gliwice, Poland) or in 4% glutaraldehyde (Avantor Performance Materials Poland S.A., Gliwice, Poland) in 0.1 M Na cacodylate buffer (Sigma-Aldrich, Saint Louis, MO, USA), dehydrated in increasing concentrations of ethanol and dried at the critical point of carbon dioxide. Samples were glued onto holders, coated in gold (Jeol JFC-1100E ion sputter [Jeol, Tokyo, Japan] or SPIMODULE Sputter Coater [Structure Probe, Inc., Chester, PA, USA], depending on sample) and examined in a Jeol JSM-5410 SEM (Jeol, Tokyo, Japan) or in a Philips XL 30 SEM (Philips Electron Optics B.V., Eindhoven, The Netherlands).

### 4.5. Anatomy of Petiole, Stem, Peduncle of CH and CL Flower Elements

The anatomy of *V. reichenbachiana* stem, peduncle, petiole and CL flowers was analyzed on acetocarmine-stained handmade cross-sections as well as on microtome-made paraffin cross-sections stained with Heidenhain’s hematoxylin combined with Alcian blue according to the procedure described in [39].

*Viola caspia* stems and peduncles were fixed in FAA (10 mL formalin [Sigma-Aldrich, Saint Louis, MO, USA], 5 mL glacial acetic acid [Avantor Performance Materials Poland S.A., Gliwice, Poland] and 85 mL 70% ethanol [Avantor Performance Materials Poland S.A., Gliwice, Poland]) for 48 h and then washed in 50% ethanol, dehydrated in a butanol series (Sigma-Aldrich, Saint Louis, MO, USA), embedded in paraffin wax (melting point 56 °C; Avantor Performance Materials Poland S.A., Gliwice, Poland), sectioned by rotary microtome (Adamas Instrumenten B.V., HM 340E, Rhenen, The Netherlands). at a thickness of 20 μm, double s tained with crystal violet–erythrosine, cleared in xylene (Avantor Performance Materials Poland S.A., Gliwice, Poland) and mounted in Canada balsam (Sigma-Aldrich, Saint Louis, MO, USA).

### 4.6. Establishing Differences in the Characteristics of V. caspia—Live Collection of Iranian Violets vs. Greenhouse Conditions

Twenty plants were cultivated in the LCIV and twenty plants were grown in pots (30 × 30 cm) containing garden soil—a mixture of sand, soil and rotten manure in equal proportions—in controlled greenhouse conditions. The photoperiod was supplied naturally according to the conditions of the season. A gradual increase in temperature and in the photoperiod from late winter to early spring as well as gradual cooling in autumn were more evident in nature than in the greenhouse, especially the gradual increase in temperature (Table S3). The data (mean parameters of daily temperature and humidity in the period of 2015–2020 in nature) were obtained in August 2021 from the Meteorological Department of Zanjan Province (http://www.zanjanmet.ir/, accessed on 27 August 2021).

Observation of CH and CL flowering and fruiting was carried out on plants growing in the greenhouse and in the LCIV throughout the season for five years (2015–2020). The number of CH and CL flowers per plant was counted in each month of the season. Every day, the open CH flowers and CL flowers were marked with a red label to avoid counting the same flowers in the following days.

The correlations (r, Pearson) between four parameters (temperature, humidity, number of CH flowers and number of CL flowers) in both conditions (greenhouse and LCIV) were counted in the stats package in R v. 3.6.3 (R Core Team, 2020).

## 5. Conclusions

(1) Environmental variables influence the timing of CH and CL flowering and the CH/CL ratio. The controlled greenhouse conditions may allow manipulation of this mixed mating system and even induce simultaneous flowering of CH and CL flowers in species with temporal separation of these two flower morphs in nature.

(2) Determination of the location of floral meristems is important for further molecular research on the differential expression of genes involved in the development of CL and CH flowers.

(3) The differences in fruit morphology between cross-pollinated CH and obligate self-pollinated CL flowers are important in population genetic diversity and in breeding programs of *Viola*.

## Supplementary Materials

The following are available online at https://www.mdpi.com/article/10.3390/plants10102147/s1. Table S1. Mean number of CH and CL per plant of *Viola caspia* in particular months growing in Live Collection of Iranian Violets (Zanjan climate). Mean from five years observation. N = 20. Table S2. Mean number of CH and CL per plant of *Viola caspia* in particular months growing in greenhouse conditions. Mean from five years observation. N = 20. Table S3. Environmental conditions in particular month in Live Collection of Iranian Violets and in greenhouse during CH and CL flowering of *Viola caspia* (mean values from years 2015–2020).

## References

[1] Kuhn M. (1867). Einige Bermerkungen über Vandelliaund den Blüten Dimorphismus. Bot. Ztg..

[2] Darwin C. (1877). The Different Forms of Flowers on Plants of the Same Species.

[3] Uphof J.C.T. (1938). Cleistogamic flowers. Bot. Rev..

[4] Lord E.M. (1981). Cleistogamy: A tool for the study of floral morphogenesis function and evolution. Bot. Rev..

[5] Culley T.M., Klooster M.R. (2007). The cleistogamous breeding system: A review of its frequency, evolution, and ecology in angiosperms. Bot. Rev..

[6] Beattie A.T. (1969). Studies in the pollination ecology of *Viola* 1. The pollen-content of stigmatic cavities. Watsonia.

[7] Elisafenko T.V. (1998). The two types of flowering in the rare Siberian species of *Viola* (Violaceae). Bot. Zhurnal.

[8] Elisafenko T.V. (2008). Ecological and biological peculiarities of *Viola prionantha* (Violaceae) under introduction. Bot. Zhurnal.

[9] Lu Y. (2002). Why is cleistogamy selected reproductive strategy in *Impatiens capensis* (Balsaminaceae)?. Biol. J. Linn. Soc..

[10] Culley T.M., Grubb T.C. (2003). Genetic effects of habitat fragmentation in *Viola pubescens* (Violaceae), a perennial herb with chasmogamous and cleistogamous flowers. Mol. Ecol..

[11] Sternberger A.L., Ruhil A.V.S., Rosenthal D.M., Ballard H.E., Wyatt S.E. (2020). Environmental impact on the temporal production of chasmogamous and cleistogamous flowers in the mixed breeding system of *Viola pubescens*. PLoS ONE.

[12] Mayers A.M., Lord E.M. (1983). Comparative flower development in the cleistogamous species *Viola odorata*. I. A growth rate study. Am. J. Bot..

[13] Mayers A.M., Lord E.M. (1983). Comparative flower development in the cleistogamous species *Viola odorata*. II. An organographic study. Am. J. Bot..

[14] Mayers A.M., Lord E.M. (1984). Comparative flower development in the cleistogamous species *Viola odorata*. III. A histological study. Int. J. Plant Sci..

[15] Geuten K., Coenen H. (2013). Heterochronic genes in plant evolution and development. Front. Plant Sci..

[16] Takahashi R., Kurosaki H., Yumoto S., Han O.K., Abe J. (2001). Genetic and linkage analysis of cleistogamy in soybean. J. Hered..

[17] Turuspekov Y., Mano Y., Honda I., Kawada N., Watanabe Y., Komatsuda T. (2004). Identification and mapping of cleistogamy genes in barley. Theor. Appl. Genet..

[18] Ning S., Wang N., Sakuma S., Pourkheirandish M., Wu J., Matsumoto T., Koba T., Komatsuda T. (2013). Structure, transcription and post-transcriptional regulation of the bread wheat orthologs of the barley cleistogamy gene Cly1. Theor. Appl. Genet..

[19] Wang Y., Ballard H.E., McNally R.R., Wyatt S.E. (2013). Gibberellins are involved but not sufficient to trigger a shift between chasmogamous-cleistogamous flower types in *Viola pubescens*. J. Torrey Bot. Soc..

[20] Wang N., Ning S., Wu J., Tagiri A., Komatsuda T. (2014). An epiallele at cly1 affects the expression of floret closing (cleistogamy) in barley. Genetics.

[21] Li Q., Huo Q., Wang J., Zhao J., Sun K., He C. (2016). Expression of B-class MADS-box genes in response to variations in photoperiod is associated with chasmogamous and cleistogamous flower development in *Viola philippica*. BMC Plant Biol..

[22] Li Q., Li J., Zhang L., Pan C., Yang N., Sun K., He C. (2021). Gibberellins are required for dimorphic flower development in *Viola philippica*. Plant Sci..

[23] Luo Y., Hu J.-Y., Li L., Luo Y.-L., Wang P.-F., Song B.-H. (2016). Genome-wide analysis of gene expression reveals gene regulatory networks that regulate chasmogamous and cleistogamous flowering in *Pseudostellaria heterophylla* (Caryophyllaceae). BMC Genom..

[24] Ballard H.E., Sytsma K.J., Kowal R.R. (1999). Shrinking the violets: Phylogenetic relationships of infrageneric groups in *Viola* (Violaceae) based on internal transcribed spacer DNA sequences. Syst. Bot..

[25] Marcussen T., Heier L., Brysting A.K., Oxelman B., Jakobsen K.S. (2015). From gene trees to a dated allopolyploid network: Insights from the angiosperm genus *Viola* (Violaceae). Syst. Biol..

[26] West G. (1930). Cleistogamy in *Viola riviniana* with especial reference to its cytological aspects. Ann. Bot..

[27] Cortes-Palomec A.C., Ballard H.E. (2006). Influence of annual fluctuations in environmental conditions on chasmogamous flower production in *Viola striata*. J. Torrey Bot. Soc..

[28] Małobęcki A., Marcussen T., Bohdanowicz J., Migdałek G., Słomka A., Kuta E. (2016). Cleistogamy and phylogenetic position of *Viola uliginosa* (Violaceae) re-examined. Bot. J. Linn. Soc..

[29] Valentine D.H., Tutin T.G., Heywood V.H., Burges N.A., Moore D.M., Valentine D.H. (1968). *Viola* L.. Flora Europaea.

[30] Marcussen T., Borgen L. (2011). Species delimitation in the Ponto-Caucasian *Viola sieheana* complex, based on evidence from allozymes, morphology, ploidy levels, and crossing experiments. Plant Syst. Evol..

[31] Marcussen T., Karlsson T., Jonsell B., Karlsson T. (2010). *Violaceae*. Flora Nordica.

[32] Khatamsaz M. (1991). *Violaceae*. Flora of Iran.

[33] Marcussen T., Borgen L., Nordal I. (2005). New distributional and molecular information call into question the systematic position of the west Asian *Viola sintenisii* (Violaceae). Bot. J. Linn. Soc..

[34] Eckstein R.L., O’Neill R.A., Danihelka J., Otte A., Köhler W. (2006). Genetic structure among and within peripheral and central populations of three endangered floodplain violets. Mol. Ecol..

[35] Migdałek G., Woźniak M., Słomka A., Godzik B., Jędrzejczyk-Korycińska M., Rostański A., Bothe H., Kuta E. (2013). Morphological differences between violets growing at heavy metal polluted and non-polluted sites. Flora.

[36] Migdałek G., Nowak J., Saługa M., Cieślak E., Szczepaniak M., Ronikier M., Marcussen T., Słomka A., Kuta E. (2017). No evidence of contemporary interploidy gene flow between the closely related European woodland violets *Viola reichenbachiana* and *V. riviniana* (sect. Viola, Violaceae). Plant Biol..

[37] Kuta E., Jędrzejczyk-Korycińska M., Cieślak E., Rostański A., Szczepaniak M., Migdałek G., Wąsowicz P., Suda J., Combik M., Słomka A. (2014). Morphological versus genetic diversity of *Viola reichenbachiana* and *V. riviniana* (sect. *Viola*, Violaceae) from soils differing in heavy metal content. Plant Biol..

[38] Paul W., Cieślak E., Ronikier M., Migdałek G., Słomka A., Żabicka J. (2016). Low genetic diversity of declining *Viola uliginosa* (Violaceae) at its southern range limits in Poland. Acta. Biol. Cracov. Ser. Bot..

[39] Kwiatkowska M., Żabicka J., Migdałek G., Żabicki P., Cubała M., Bohdanowicz J., Słomka A., Jędrzejczyk-Korycińska M., Sliwinska E., Sychta K. (2019). Comprehensive characteristics and genetic diversity of the endemic Australian *Viola banksii* (section Erpetion, Violaceae). Aust. J. Bot..

[40] Żabicka J., Migdałek G., Słomka A., Sliwinska E., Mackiewicz L., Keczyński A., Kuta E. (2020). Interspecific hybridization and introgression influence biodiversity—Based on genetic diversity of Central European *Viola epipsila*-*V. palustris* complex. Diversity.

[41] Saeidi Mehrvarz S., Vafi M., Marcussen T. (2013). Taxonomic and anatomical notes on *Viola* sect. Viola (Violaceae) in Iran. Wulfenia.

[42] Gharari Z., Sharafi A., Bagheri K., Yazdinezhad A., Bijan S. (2019). *In vitro* regeneration and secondary metabolites of *Viola caspia* subsp. sylvestrioides Marcussen. BioTechnologia.

[43] Zargari A. (1997). Medicinal Plants.

