# Supplementary material for: Seasonal and Simultaneous Cleistogamy in Rostrate Violets (Viola, subsect. Rostratae, Violaceae)
Source: Plants (Basel). 2021 Oct 10;10(10):2147. doi: 10.3390/plants10102147 (PMC8537809; doi:10.3390/plants10102147)
Supplement: Supplementary file 1 [file plants-10-02147-s001.zip › plants-1377628-supplementary.pdf]

Table S1. Mean number of CH and CL per plant of *Viola caspia* in particular months growing in Live Collection of Iranian Violets (Zanjan climate). Mean from five years observation. N = 20.

CH flowers												
Year	January	February	March	April	May	June	July	August	September[a]	October	November	December
2016	1	1	18	8	2	0	0	0	0	0	0	0
2017	1	2	17	10	2	0	0	0	0	0	0	0
2018	0	2	19	7	3	0	0	0	0	0	0	0
2019	2	3	18	6	2	0	0	0	0	0	0	0
2020	1	2	**18**	9	1	0	0	0	0	0	0	0
Mean	1	2	18	8	2	0	0	0	0	0	0	0
CL flowers												
2016	0	0	0	3	6	0	0	0	0	0	0	0
2017	0	0	1	5	5	0	0	0	0	0	0	0
2018	0	0	1	5	5	0	0	0	0	0	0	0
2019	0	0	2	4	4	0	0	0	0	0	0	0
2020	0	0	1	3	5	0	0	0	0	0	0	0
Mean	0	0	1	4	5	0	0	0	0	0	0	0
Mean CH/CL ratio	0	0	18	2	0.4	0	0	0	0	0	0	0

[a] The plants gradually begin to fall from this month and are dormant in winter until January.

Table S2. Mean number of CH and CL per plant of *Viola caspia* in particular months growing in greenhouse conditions. Mean from five years observation. N = 20.

CH flowers												
Year	January	February	March	April	May	June	July	August	September	October	November	December
2016	17	20	23	16	12	0	0	0	0	0	0	0
2017	16	19	23	17	12	3	0	0	0	0	0	0
2018	16	18	22	17	10	2	0	0	0	0	0	0
2019	15	18	22	15	8	3	0	0	0	0	0	0
2020	16	19	24	14	8	2	0	0	0	0	0	0
Mean	16	19	**22**	16	10	2	0	0	0	0	0	0
CL flowers												
2016	10	13	11	13	11	8	6	0	1	0	4	7
2017	10	11	14	13	11	8	5	2	0	1	4	6
2018	8	12	12	12	11	6	7	1	0	1	4	7
2019	9	12	13	14	10	7	2	3	1	2	7	7
2020	8	12	10	13	7	6	5	4	3	1	6	8
Mean	9	12	**14**	13	10	7	5	2	1	1	5	7
Mean CH/CL ratio	1.77	1.58	1.57	1.23	1.0	0.28	0	0	0	0	0	0

Table S3. Environmental conditions in particular month in Live Collection of Iranian Violets and in greenhouse during CH and CL flowering of *Viola caspia* (mean values from years 2015-2020).

	Live Collection of Iranian Violets		Greenhouse	
Month	Temp. [°C] daily	Humidity [%]	Temp. [°C] daily	Humidity [%]
January	5.8	48.4	20.5	60.1
February	2.1	65.2	21.8	62.2
March	0.4	62	22.5	65.1
April	1.7	63	22.7	55.2
May	7.2	56	24.3	54.5
June	10.0	56	24.4	54.3
July	16.7	52.4	24.0	49.2
August	22.4	42	23.4	50.2
September	25.8	43.7	21.6	54.1
October	24.3	35	20.8	56.5
November	20.1	41.9	20.5	62.1
December	13.1	50.4	20.4	65.2
